# Influência da tomografia computadorizada no planejamento cirúrgico das fraturas do maléolo posterior

**DOI:** 10.1055/s-0045-1810041

**Published:** 2025-09-08

**Authors:** Noé De Marchi Neto, Pietro Felice Tomazini Nesello, Jordanna Maria Pereira Bergamasco, Marco Túlio Costa, Ralph Walter Christian, Nilson Roberto Severino

**Affiliations:** 1 Departamento de Ortopedia e Traumatologia da Santa Casa de São Paulo – Pavilhão Fernandinho Simonsen, Faculdade de Ciências Médicas da Santa Casa de São Paulo, São Paulo, SP, Brasil.

**Keywords:** cirurgiões, fraturas do tornozelo, radiografia, tomografia computadorizada por raios x, ankle fractures, radiography, surgeons, tomography, x-ray computed

## Abstract

**Objetivo:**

Este estudo teve como objetivo avaliar a influência da tomografia computadorizada (TC) no planejamento pré-operatório das fraturas do maléolo posterior (MP) do tornozelo, comparando suas informações com as de radiografias convencionais (RX) e o seu impacto no tratamento cirúrgico.

**Métodos:**

A pesquisa incluiu 81 pacientes com fraturas do MP, cujas imagens de RX e TC foram analisadas por 33 cirurgiões ortopédicos especializados. O estudo foi dividido em duas etapas, com avaliação de RX na primeira e de RX e TC na segunda. Em ambas as etapas, perguntou-se o tamanho do MP, a estabilidade da fratura, a conduta pré-operatória e as possíveis modificações após avaliar a TC.

**Resultados:**

Os resultados mostraram que, ao considerar apenas o RX, 83,5% dos avaliadores decidiram pela fixação do MP. No entanto, ao adicionar a TC, essa decisão foi modificada em 49,1% dos casos, influenciando a via de acesso cirúrgico e o tipo de osteossíntese. Em 34,7% dos casos, a TC revelou um fragmento maior do MP em comparação ao RX, demonstrando sua superioridade na avaliação do tamanho e morfologia da fratura.

**Conclusão:**

A TC deve ser incluída de forma rotineira no planejamento cirúrgico das fraturas do tornozelo com comprometimento do maléolo posterior, pois permite uma avaliação mais precisa do traço de fratura e pode mudar a decisão terapêutica baseada somente na RX.

## Abstract (en)

**Objective:**

The present study aimed to evaluate the influence of computed tomography (CT) on the preoperative planning of posterior malleolus (PM) fractures of the ankle, comparing its information with that of conventional radiographs and assessing its impact on surgical treatment.

**Methods:**

The study included 81 patients with PM fractures, whose radiological and CT images were analyzed by 33 specialized orthopedic surgeons. The study had two stages, with a radiological assessment on the first, and the second having radiological and CT evaluation. In both stages, we asked surgeons about the PM size, fracture stability, preoperative management, and potential modifications after CT analysis.

**Results:**

Considering only radiographs, 83.5% of the evaluators selected PM fixation. However, CT addition modified this choice in 49.1% of the cases, influencing the surgical access route and the type of osteosynthesis. In 34.7% of cases, CT revealed a larger PM fragment than radiographs, demonstrating that it is superior in evaluating fracture size and morphology.

**Conclusion:**

The surgical planning of ankle fractures with PM involvement should routinely include CT scans for a more precise fracture line assessment and a potential change in the therapeutic decision based on simple radiography alone.

## Introdução

As fraturas do tornozelo são frequentes na prática dos ortopedistas. Aproximadamente 40% das maleolares acometem o maléolo posterior (MP). Esse tipo de fratura está associado a maior instabilidade e incongruência articular, maior complexidade na redução articular e maior risco de desenvolver osteoartrose a longo prazo. 1 2

Durante muitos anos, o tratamento das fraturas do maléolo posterior (MP) foi baseado no tamanho da fratura em relação à articulação tíbio-talar, avaliado nas radiografias (RX) pré-operatórias. 3 No entanto, desde o final do século XX, a avaliação RX do MP tem sido criticada, pois muitas vezes não esclarece adequadamente a complexidade da fratura ou subestima a dimensão do fragmento posterior. 2 4 5 6 A partir dos anos 2000, a tomografia computadorizada (TC) passou a ser uma ferramenta valiosa na avaliação das fraturas do MP, permitindo uma interpretação mais precisa dos padrões de fraturas e auxiliando na programação pré-operatória onde novas classificações foram criadas exclusivamente para o fragmento posterior. 7 8 9

A TC pré-operatória mostra um tamanho maior do MP quando comparado com o RX 10 e traz mais informações quanto aos traços de fratura, afundamento articular, fragmentos interpostos; além de mostrar a relação do próprio MP com os outros maléolos e a sindesmose. Atualmente, a TC em conjunto com RX é cada vez mais recomendada no planejamento cirúrgico das fraturas articulares do tornozelo. 5 11 12

O objetivo desse estudo é avaliar o impacto da TC na avaliação pré-operatória da fratura do tornozelo e o que as informações obtidas com a TC influenciariam na interpretação do MP e na conduta tomada pelo cirurgião ortopédico.

## Materiais e Métodos

Em um período de 5 anos, entre 2016 e 2021, 144 pacientes com diagnóstico de fratura do tornozelo envolvendo MP foram tratados no Centro de Trauma Nível 1 do nosso hospital. Foram selecionados para o estudo casos de fraturas ou fraturas-luxação do tornozelo que acometiam o MP de pacientes acima de 18 anos e com documentação adequada de RX e TC. Casos com fraturas do pilão tibial, fraturas de tornozelo associadas à outras fraturas do retropé e pacientes com esqueleto imaturo foram excluídos. Esse estudo foi cadastrado e aprovado pelo Comitê de Ética e Pesquisa sob o CAAE número 52916921.1.0000.5479.

**Fig. 1:**
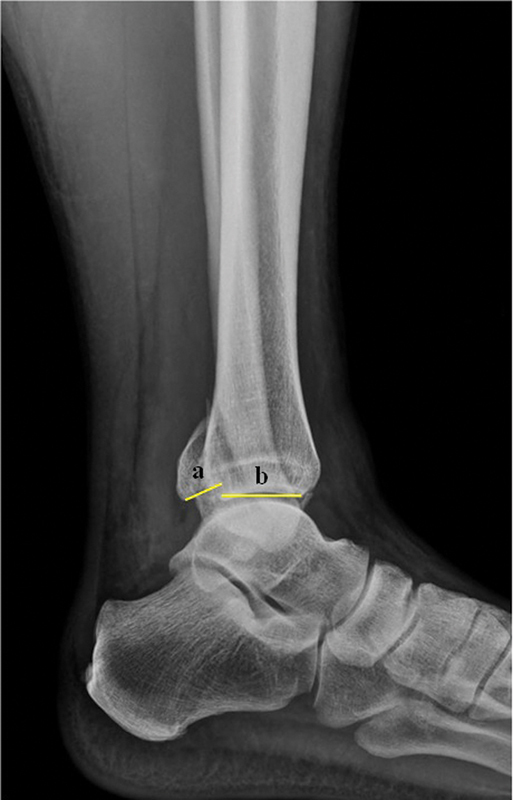
Medida do tamanho do fragmento de fratura do maléolo posterior na radiografia em perfil. Aferiu-se a proporção entre a fratura e a totalidade da superfície articular, sendo a relação (a)/(a + b).

O estudo incluiu 81, que foram pré-avaliados por dois ortopedistas da nossa instituição. Ambos são especialistas em cirurgia de pé e tornozelo que, de forma independente, aferiram as dimensões do MP nos exames de imagem ( Figura 1 ) e classificaram as fraturas segundo Haraguchi. 7 A média do tamanho percentual do fragmento do MP em relação a superfície articular total foi definida como valor padrão dos casos bem como a classificação tomográfica para a montagem do estudo.

Com base nos valores padrão, foram selecionados casos para formar conjuntos de 10 fraturas do maléolo posterior com diferentes tamanhos conforme evidenciado nas radiografias. Os intervalos considerados foram < 15,0%; 15,0 a 19,9%; 20,0 a 24,9%; 25,0 a 29,9% e > 30,0% da superfície articular total, além de abranger diferentes tipos tomográficos de fraturas do MP.

O estudo foi feito em duas etapas sendo que a ordem dos casos apresentados para os avaliadores foi sorteada previamente para cada etapa ( Tabela 1 ).

**Tabela 1:** Importância do MP na estabilidade da fratura do tornozelo de acordo com seu tamanho na radiografia

Casos	Estabilidade	Média	DP	IC 95% inferior	IC 95% superior	Valor de *p*
< 15%						
	Não	79,4%	30,4%	68,6%	90,2%	< 0,01*
	Sim	20,6%	30,4%	9,8%	31,4%	
15–19,9%						
	Não	45,6%	39,6%	31,5%	59,6%	< 0,01**
	Sim	54,4%	39,6%	40,4%	68,5%	
20–24,9%						
	Não	44,1%	29,6%	33,6%	54,6%	< 0,01***
	Sim	55,9%	29,6%	45,4%	66,4%	
25–29,9%						
	Não	11,8%	30,3%	1,0%	22,5%	< 0,01***
	Sim	88,2%	30,3%	77,5%	99,0%	
> 30%						
	Não	14,7%	35,9%	2,0%	27,5%	NA
	Sim	85,3%	35,9%	72,5%	98,0%	

**Abreviações:** DP, desvio padrão; IC, intervalo de confiança; MP, maléolo posterior.

**Notas:** * Comprado a todas as outras categorias de MP. ** Comparado às categorias 25–29,9% e ≥ 30%. *** Comparado a > 30%.

**Fig. 2:**
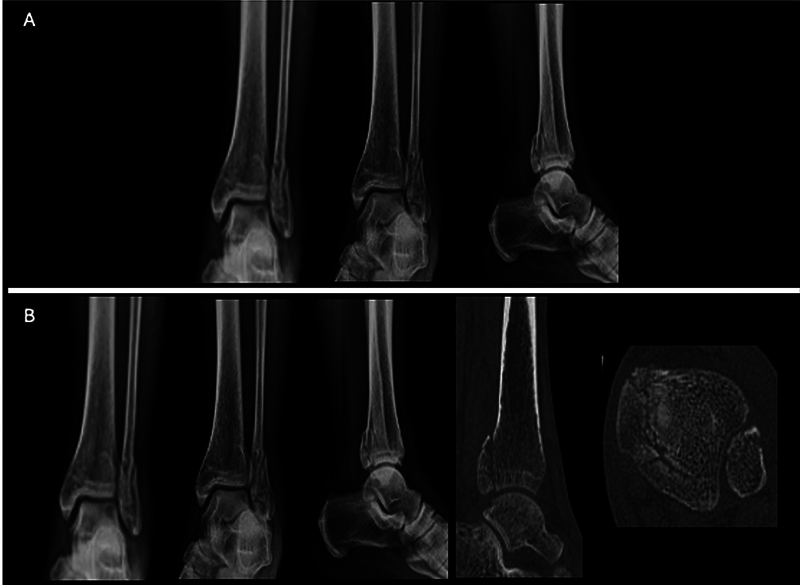
Exemplo de caso enviado para cada avaliador. A 1ª etapa ( **A** ) exclusivamente com RX e ( **B** ) contendo RX, TC em corte sagital com o maior fragmento do MP e TC corte axial 5 mm acima da superfície articular. **Abreviaturas:** MP, maléolo posterior; RX, radiografia; TC, tomografia computadorizada.

Na primeira etapa (E1), os casos foram organizados em ordem alfabética de A a J, utilizando radiografias do tornozelo nas incidências anteroposterior e lateral, com ênfase na fratura MP. Na segunda etapa (E2), os mesmos casos foram reorganizados de Q a Z, apresentando as mesmas radiografias da E1, agora complementadas por imagens tomográficas em cortes sagitais e axiais, que detalhavam a fratura ( Figura 2 ).

Foram convidados 40 médicos ortopedistas de diferentes hospitais para participar como avaliadores. Todos os que aceitaram preencheram um termo de consentimento, previamente aprovado pelo Comitê de Ética Médica da instituição. Para participar, era necessário que cada avaliador fosse especialista com formação em cirurgia de pé e tornozelo, vinculado a um Serviço de Ensino de Residência Médica em Ortopedia e Traumatologia ou a um Centro Formador de Especialistas em Cirurgia de Pé e Tornozelo credenciado pelas respectivas Sociedades.

Cada avaliador recebeu, por e-mail, um conjunto de 10 casos e respondeu individualmente às duas etapas da pesquisa, com um intervalo de 2 meses entre elas. Para cada caso, eram fornecidas informações sobre idade, sexo e mecanismo de trauma (entorse do tornozelo, queda de altura, acidente de trânsito, trauma durante prática esportiva). Além disso, cada conjunto incluía um questionário padronizado. Na E1, as perguntas abordavam exclusivamente as imagens radiográficas, enquanto na E2, as questões eram direcionadas tanto para o RX quanto para a TC ( **Anexo 1–Questionário** ).

Em ambas as etapas, as perguntas relacionadas às radiografias abordavam aspectos como a estabilidade da fratura, interpretação do tamanho do MP, indicação de fixação e o tipo de fixação recomendada. Já as questões sobre TC incluíam comparações do tamanho do MP em relação ao RX, classificação tomográfica, possíveis alterações na conduta, indicação de fixação e o tipo de síntese a ser utilizada.

Após a conclusão das duas etapas, foram comparadas as respostas das radiografias de E1 e E2 com o objetivo de avaliar a concordância intra- e interobservador em dois momentos distintos. As respostas referentes à TC foram analisadas para verificar a precisão da classificação tomográfica pelos avaliadores, além de serem comparadas com as respostas dadas para as questões radiográficas. Por fim, foi avaliado quanto a TC influenciou na interpretação da fratura e na definição da conduta pré-operatória.

## Análise Estatística

A análise dos dados foi realizada utilizando o software Statistical Package Social Sciences (SPSS, IBM Corp.) versão 25 para MAC. Os dados categóricos foram descritos por suas frequências absolutas e relativas. A concordância intra- e interavaliadores foi avaliada pelos testes Kappa e McNemar.

Os dados contínuos ou numéricos foram descritos pela média e desvio padrão (DP), e, quando apropriado, pela mediana e pelos percentis 25 e 75. A normalidade dos dados contínuos foi testada por meio do teste de Shapiro-Wilk. Para a comparação entre as proporções das mudanças de conduta, foram empregados testes não paramétricos: o de Mann-Whitney para amostras independentes e o de Friedman para múltiplas comparações.

Em todas as análises estatísticas, foi considerado um nível de significância de *p* < 0,05.

## Resultados

O estudo contou com a participação de 33 avaliadores com uma média de idade de 45 ± 8 anos. O grupo tinha uma média de 15 ± 7 anos de experiência em cirurgia de pé e tornozelo e tratavam, em média, 65 (39–86) casos/ano de fraturas de tornozelo.

## Tamanho do Maléolo Posterior

Na análise das radiografias, em média, 59,7 ± 15,3% dos avaliadores estimaram o tamanho do MP de forma semelhante aos valores dos subgrupos padrão, com índices de concordância de 0,16 e 0,15, respectivamente, nas duas etapas. Nas etapas E1 e E2, 78,8 ± 19,9% dos avaliadores consideraram o tamanho do MP relevante para a estabilidade das fraturas, e sua importância aumentou proporcionalmente ao tamanho do fragmento, com um índice de concordância de 0,21 ( *p* < 0,01), como pode ser visto na Tabela 1 .

Considerando exclusivamente a tomografia, a concordância dos avaliadores referente à classificação de Haraguchi 7 foi de 66,1 ± 4,82%. Além disso, em 60,9 ± 15,8% dos casos os avaliadores assinalaram um tamanho do MP na TC semelhante ao que haviam medido no RX.

**Fig. 3:**
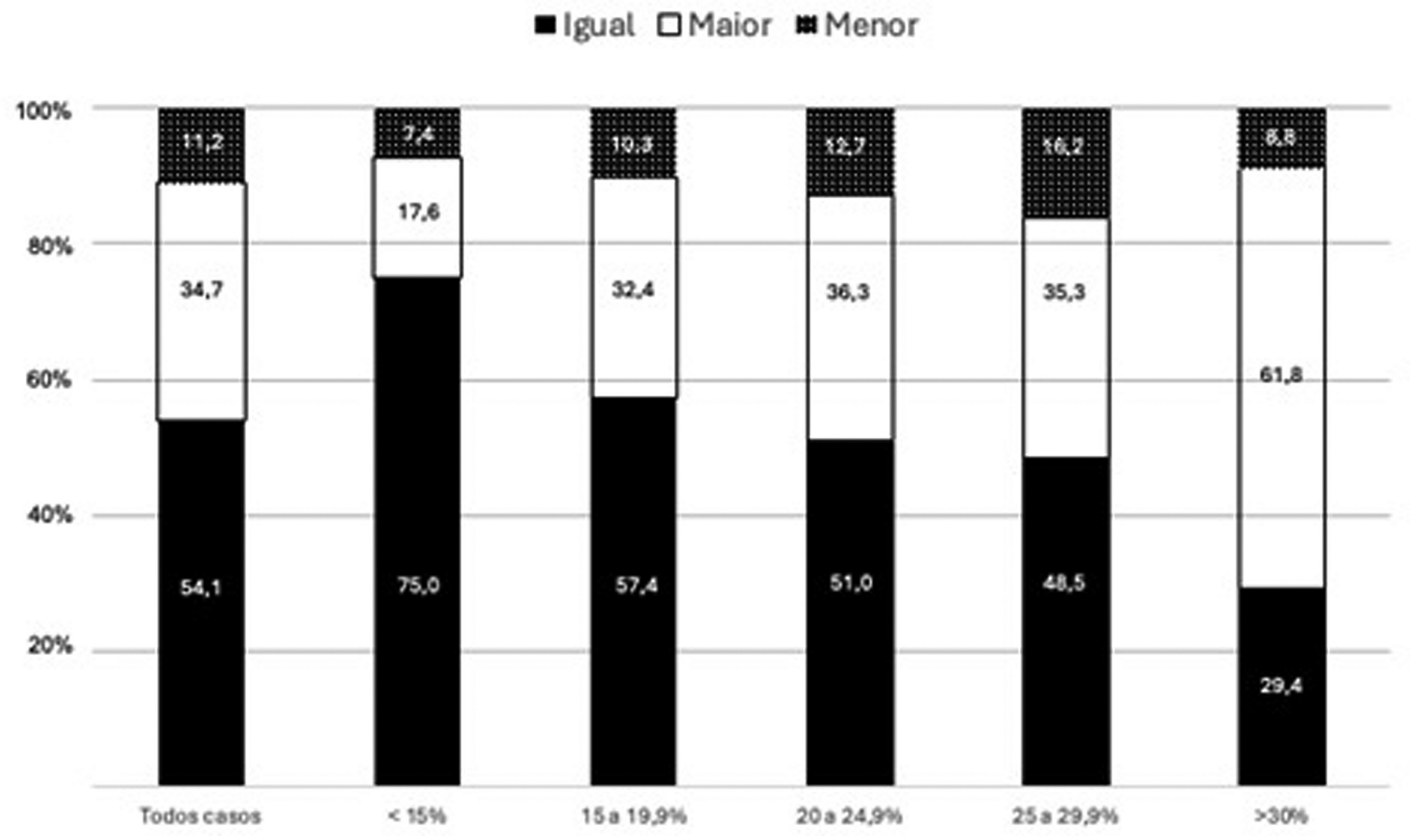
Interpretação dos avaliadores ao comparar o tamanho do maléolo posterior nas radiografias com a tomografia.

Quando o tamanho do MP foi comparado simultaneamente nas RX e na TC, 54,1% dos avaliadores consideraram os tamanhos semelhantes em ambas as modalidades. Em 34,7% dos casos, o MP foi interpretado como maior na TC em relação ao RX, enquanto em 11,2% o MP parecia menor na TC. A Tabela 2 e a Figura 3 ilustram as diferenças nos tamanhos do MP entre os subgrupos. Observa-se que, à medida que o tamanho aumenta, maior é a divergência na interpretação entre as imagens de RX e TC.

**Tabela 2:** Comparação do tamanho do MP na TC com o previamente aferido no RX na totalidade dos casos e em cada intervalo

	Igual		Maior		Menor		
Tamanho MP no RX	Média	DP	Média	DP	Média	DP	Valor de *p*
**Total**	54,1%	16%	34,7%	16%	11,2%	11%	N/D
**< 15%**	75,0%	30,8%	17,6%	29,9%	7,4%	18,0%	< 0,05*
**15–19,9%**	57,4%	35,1%	32,4%	32,3%	10,3%	23,9%	< 0,05**
**20–24,9%**	51,0%	33,1%	36,3%	30,0%	12,7%	16,4%	> 0,05***
**25–29,9%**	48,5%	33,7%	35,3%	33,8%	16,2%	29,4%	N/D
**> 30%**	29,4%	46,2%	61,8%	49,3%	8,8%	28,8%	< 0,05*

**Abreviações:** DP, desvio padrão; MP, maléolo posterior; N/D, não disponível; RX, radiografia; TC, tomografia computadorizada.

**Notas:** * Comparado a todas as categorias de MP. ** Comparado às categorias 20–24,9% de MP e 25–29,9%%. *** Comparadoà categoria 25–29,9% de MP.

## Tratamento do Maléolo Posterior

Os avaliadores concordaram em 83,5 ± 11% em relação a conduta de fixar (ou não) o MP nas 2 etapas avaliando apenas as radiografias, com concordância de 0,36 e 0,34, respectivamente.

**Fig. 4:**
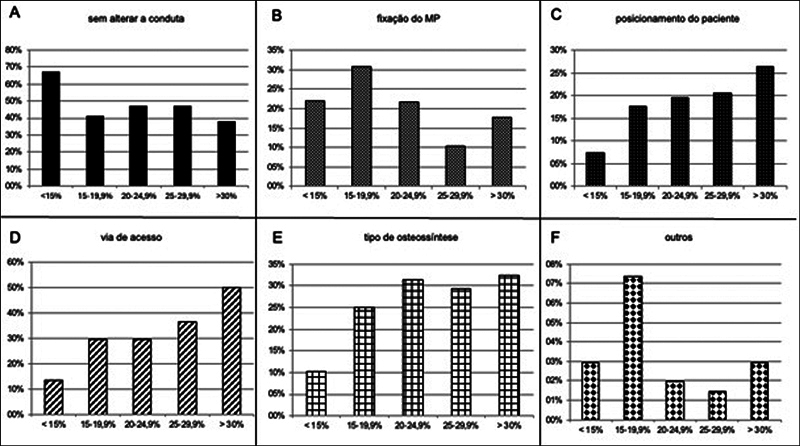
Alterações de conduta dos avaliadores após avaliar RX e TC em conjunto. ( **A** ) Sem mudança na conduta, ( **B** ) fixação do MP após ver a TC, ( **C** ) posicionamento do paciente no ato cirúrgico, ( **D** ) via de acesso cirúrgico, ( **E** ) tipo de osteossíntese escolhida para fixação, ( **F** ) outras modificações. **Abreviaturas:** MP, maléolo posterior; RX, radiografia; TC, tomografia computadorizada.

Na análise simultânea dos RX e TC, em 49,1% dos casos, não haveria mudança na conduta dos avaliadores. Nos demais casos, a TC influenciou a decisão clínica, modificando a via de acesso em 29,7%, o tipo de osteossíntese em 25,6%, a indicação para fixação do MP em 20,9% e a posição de decúbito do paciente em 17,6%. A Tabela 3 apresenta as modificações de conduta sugeridas em cada subgrupo, e a Figura 4 ilustra essas mudanças. Nos grupos com fraturas envolvendo menos de 25% do MP, a alteração mais frequente foi a indicação de fixação. Já nas fraturas com fragmentos maiores que 25%, houve um aumento progressivo nas mudanças de conduta relacionadas à via de acesso, posicionamento e escolha da osteossíntese.

**Tabela 3:** Alterações de conduta dos avaliadores nos diferentes intervalos de tamanho do MP após analisar a TC em conjunto com o RX

Tamanho MP	Nada	Fixar o MP	Posicionamento do paciente	Via de acesso	Tipo de osteossíntese	Outro	
< 15%	67,6%	22,1%	7,4%	13,2%	10,3%	2,9%	
15–19,9%	41,2%	30,9%	17,6%	29,4%	25,0%	7,4%	
20–24,9%	47,0%	21,5%	19,6%	29,4%	31,3%	1,9%	
25–29,9%	47,0%	10,3%	20,5%	36,7%	29,4%	1,4%	
> 30%	38,2%	17,6%	26,5%	50,0%	32,4%	2,9%	

**Abreviação:** MP, maléolo posterior; RX, radiografia; TC, tomografia computadorizada.

## Discussão

A TC é amplamente utilizada no planejamento pré-operatório das fraturas do MP devido a três principais fatores. Primeiro, ela permite visualizar com precisão o tamanho real do fragmento posterior. 10 Segundo, fraturas que se estendem além da porção posterolateral da tíbia, fraturas multifragmentadas ou com fragmentos interpostos não podem ser adequadamente avaliadas apenas por radiografias. 13 14 Por fim, a TC auxilia o cirurgião na escolha da via de acesso, posicionamento do paciente e seleção dos implantes de fixação. 12 15 Atualmente, muitos autores recomendam seu uso rotineiro no pré-operatório em fraturas de tornozelo. 5 16

Neste estudo, a análise das imagens radiográficas revelou uma variação significativa na precisão dos avaliadores ao medir o tamanho do MP, com uma média de 59,7 ± 15,3% em relação aos valores padrão. Embora essa média seja considerada satisfatória, a concordância foi baixa, com índices de 0,16 e 0,15 nas duas etapas. Esses resultados estão alinhados com outras publicações que também relatam baixa concordância entre avaliadores na análise de parâmetros radiográficos para fraturas do MP. 17

A relevância do MP para a estabilidade das fraturas foi enfatizada pela maioria (78,8 ± 19,9%) dos avaliadores nas etapas E1 e E2. Esse reconhecimento aumenta conforme o tamanho do MP cresce, o que é esperado, já que fragmentos maiores que 25% são tradicionalmente indicados para fixação. No entanto, fragmentos menores que 25% também estão sendo cada vez mais fixados, visando melhorar a estabilidade articular. Embora o estudo tenha destacado a importância do tamanho do MP, a literatura recente enfatiza que sua morfologia e redução correta são fatores cruciais para o planejamento do tratamento. 18 19 20

Nas imagens de TC, os avaliadores apresentaram uma taxa média de acerto de 66,1 ± 4,82% ao utilizar a classificação de Haraguchi. 7 De modo geral, estudos comparativos das principais classificações tomográficas mostram resultados semelhantes aos desta série. 21 22 23 A classificação de Haraguchi foi escolha para o presente estudo por ser a primeira descrita e amplamente utilizada até hoje, frequentemente em conjunto com a classificação de Bartoníček. 8 21 22

Quando o tamanho do MP foi comparado entre radiografias e TC, observou-se concordância em 54,1% dos casos. Entretanto, em 34,7%, o tamanho foi maior na TC. Essa diferença aumentou à medida que o tamanho do MP crescia, reforçando a hipótese de que a TC proporciona uma análise tridimensional mais precisa das fraturas, especialmente nas mais complexas. 5 13 14 15 A crescente divergência entre as medições de RX e TC à medida que o MP aumenta sugere que a TC deve ser recomendada, mesmo para fragmentos maiores, com possíveis implicações diretas na escolha do tratamento. 11

Na avaliação do tratamento da fratura posterior, os avaliadores mostraram alta concordância na decisão de fixar ou não o MP com base apenas nas radiografias (83,5%, com índice de concordância de 0,36). Entretanto, a introdução da TC modificou a conduta em 49,1% dos casos, influenciando a escolha da via de acesso (29,7%), o tipo de osteossíntese (25,6%) e a indicação de fixação do MP (20,9%). Esses resultados reforçam a relevância da TC na avaliação pré-operatória e no planejamento do tratamento. Gibson et al. demonstraram que ela altera significativamente os planos cirúrgicos em fraturas trimaleolares, com mudanças na técnica operatória em 25,1% dos casos, e uma tendência em 16,3% para optar pela fixação após a análise da TC. 24

No tratamento das fraturas do MP com tamanho inferior a 25%, a principal mudança foi o aumento na indicação de fixação, sugerindo que o RX pode subestimar a importância do MP na estabilidade da fratura, e que a TC poderia oferece uma avaliação mais detalhada, revelando o tamanho real da lesão e eventuais desvios articulares. 5 12 15 Em fraturas com fragmentos maiores que 25%, o tamanho por si só geralmente justifica a fixação, mas a análise pela TC resultou em mudanças significativas na abordagem, incluindo o posicionamento do paciente, a via de acesso cirúrgico e o tipo de osteossíntese. Esses ajustes permitiram uma interpretação mais precisa do fragmento do MP pelos avaliadores. 25

Este estudo é pioneiro na literatura nacional e se baseia em uma longa série de casos avaliados por 33 médicos experientes na área. No entanto, apresenta algumas limitações. Por se tratar de um estudo retrospectivo focado exclusivamente no fragmento do MP, não foram incluídas dados clínicos ou de exame físico dos pacientes. Além disso, como o objetivo foi investigar apenas a interpretação pré-operatória do MP, o estudo não aborda o tratamento de cada caso nem os resultados funcionais a longo prazo, o que limita a análise de desfechos clínicos e terapêuticos.

## Conclusão

O estudo reforça a importância da TC no planejamento pré-operatório das fraturas do MP. Essa técnica de imagem possibilita uma análise mais precisa especialmente em fraturas complexas e multifragmentadas, sendo fundamental na decisão de fixação para fragmentos menores que 25%.

Além disso, o uso da TC permite, mesmo nas fraturas maiores que 25%, ajustes significativos no posicionamento do paciente, escolha da via de acesso e tipo de osteossíntese, mostrando seu impacto direto no planejamento cirúrgico.
